# Metritis

**Published:** 1887-02

**Authors:** Edward B. Weston

**Affiliations:** 3225 Vernon avenue


					﻿THE CHICAGO
Medical Journal and Examiner
Vol. LIV.	FEBRUARY, 1887.	No. 2.
OKIGINAU (sOMMUNIGATIONS.
Metritis.
By Edward B. Weston, M.D.
[Candidate’s Thesis, Read before the Chicago Gyntecological Society, December 17th, 1886.]
In studying the history and literature of any medical sub-
ject we are forcibly reminded of the statement made by Aris-
totle two thousand years ago, that “ probably all art and all
wisdom have often been already fully explored and again
quite forgotten.”
In selecting “ Metritis” for my thesis, it is not for the pur-
pose of bringing to your attention “something fully explored
and quite forgotten,” or something new, but to ask your indul-
gence while the subject is briefly reviewed.
It is a subject as old as medicine,and often learnedly treated ;
yet concerning which there is still far from unanimity of
opinion.
Whether metritis is the best name for the disease depends
upon its pathology, which we do not attempt to indicate by
its use. We use it for convenience. Many gynaecologists have
attempted to formulate their opinion of its pathology by the
use of a distinguishing name ; but as yet none has been pro-
posed which is satisfactory to all.
Metritis—literally an inflammation of the womb—would, if
strictly considered, denote an inflammation of all parts of the
organ, peritoneal, muscular and mucous.
If it were so used it would come nearer to indicating its path-
ology. But the term as employed indicates a pathological
condition—acute or chronic—of the parenchyma of the uterus.
Some have considered a cervical and corporeal metritis ; but,
as the cases in which one part alone is involved are very rare,
we shall ignore this subdivision, without denying that one part
may be affected without the other, and stating that the neck
or body is primarily and chiefly involved, as the r
Acute Metritis—puerperal and non-puerperal. That uncom-
plicated acute non-puerperal metritis is a rare disease, all ad-
mit. Thomas, in an early edition of his “ Diseases of Women,”
says he had observed it but twice. Others of broad observa-
tion have never seen a case. Emmet says : “ There is no proof
that the uterine tissue adjacent to the lining membrane is ever
involved, except in the puerperal state.” Goodell writes:
“ As acute metritis, apart from child-birth, rarely exists,
excepting as an advanced state of acute endometritis, I
shall make no further allusion to it than the present one.”
Byford does not consider it so rare as most authors do ; and a
great majority of the profession believe that the disease is
occasionally met with.
When the parenchyma has become inflamed the mucous
membrane rarely altogether escapes. Indeed, when wp con-
sider the causes of the disease this could hardly be possible,
and in cases where there is no doubt as to the propriety of
designating them metritis, there was probably first a mild en-
dometritis. When the disease arises from an operation on, or
injury to, some neighboring part, as the vagina or bladder, we
can conceive of its being pure and uncomplicated, the cause
being conveyed by blood or lymph vessels directly to the part,
and that if the disease is mild and of short duration other
structures may escape. But if the disease has arisen from an
intra-uterine or vaginal pessary, the use of the sound or any
operation on the organ itself, the disease probably starts in the
mucous membrane, and the metritis, though greatly over-
shadowing the endometritis, is a secondary disease. At any
rate a wound has been made in the mucous membrane through
which the cause has at least passed. Indeed, it is now denied
that a true inflammation of the connective tissue of the uterus
is possible, except where a wound exists, and also a septic
material, to gain entrance through the wound. An operation
may be performed and primary union occur without metritis
developing; and, again, the septic material may be present and
an intact membrane bar its entrance. If the wound and poison
both exist, a metritis will be developed, and the disease prob-
ably progress until neighboring structures are involved and
the case ceases to be a simple uncomplicated one. Sudden
suppression of the menses, intemperate coitus, tumors in or
near the uterus are also among the causes.
After child-birth the uterus becomes inflamed much more
frequently. If the accoucheur has been too meddlesome, made
too frequent and rough examinations, used instruments un-
skilfully, removed the placenta violently or but partially, the
vagina cervix internal surface of the uterus may have
been seriously wounded, and the channel formed through
which the readily decomposing discharges may gain entrance
to the organ. If proper antiseptic precautions were not ob-
served both during labour and after delivery we shall probably
have metritis.
In acute non-puerperal metritis the uteris is enlarged, chiefly
antero-posteriorly; it may be even to its size in the second or
third month in pregnancy. “ On section the muscular wall is
thickened, but soft and pulpy, and yields on compression a
yellowish-red exudation. The mucous membrane is thick-
ened and vascular, but the cavity of the ute*rus is not altered
in size. Microscopically the muscular bundles are infiltrated
with pus corpuscles.” (Hart and Barbour).
The symptoms are not pathognomonic. There is fever,
usually proportionate to the amount of local disease, and in
most cases there are chills at the beginning. Severe pain
is experienced which often extends down the thighs, and to
the bladder and rectum, the evacuation of which greatly
increases the suffering. There may be diarrhoea, nausea and
vomiting. On examination, we find tenderness over the hypo-
gastric region. On introducing the finger into the vagina it will
be found at first hot and dry, the uterus usually low, swollen,
very sensitive, but movable unless old adhesions exist. Rec-
tal and bi-manual examination, if it can be borne, will aid us
in our diagnosis. After the disease has lasted a few days
the fever and pain gradually decrease, and a leucorrhceal
discharge shows that there is a complicating endometritis.
Few would feel themselves competent to say that they
had before them a pure case of metritis, without the slightest
complication. However, if the uterus is movable, and no
peri-uterine deposit be found, and if the pain and tenderness
do not seem to extend from the uterus, we may conclude that
it is chiefly affected. The disease may terminate in resolu-
tion, or in the formation of an abscess, which may burst into
the uterus itself, the bladder, the vagina, the intestines or
even through the abdominal walls. But the most common
termination is in chronic metritis. Some, however deny
that it ever so ends.
The prognosis must be guarded, as the outcome will de-
pend on which course the disease pursues.
Treatment.—We must obtain for the patient as far as pos-
sible, perfect rest. Pain must be relieved, and in most cases it
will be necessary to at once administer opium in some form.
Milder remedies usually fail and need not be experimented
with. Morphine, hypodermically, is usually best, as medi-
cine taken into the stomach is very apt to nauseate, and ano-
dyne rectal injections for a time increase the pain by their
pressure, even though it be slight. A rectal suppository is
preferable to an injection. It is well if the rectum can be
kept empty. Some advise cathartics; others, enemata. The
pain caused by the pressure of the latter on the uterus leads
me to prefer a suitable cathartic. Vaginal injections of warm
water, containing opium, given from a fountain syringe,
.gently and with little force, may be used daily or oftener.
The openings in the syringe-pipe should all open or point
backward. The patient being on her back the water will
sufficiently distend the vagina and come in contact with every
part of it, and the danger of forcing water into the uterus
unintentionally be reduced to the minimum. It is often ad
vised to use a pipe without a terminal orifice. But there is
more danger from the lateral openings, if the uterus is in its
normal position, or anteverted or strongly retroverted. Poul-
tices of flaxseed meal, made much lighter than they usually
are, and covered with oiled silk, can be borne in most cases.
If they cannot, two thicknesses of flannel, wrung out of
hot water, and covered with waterproof material, may be used.
If the case is severe, blood may be drawn, by scarifying the
uterus, or by leeches applied to the perineum or abdomen.
This should not be overdone, for in nothing is it more im-
portant to observe the golden mean. Ice cold applications to
the abdomen have been recommended. In a case caused by
a portion of retained placenta, or where it is feared the uterus
may contain septic matter, it must be at once rendered aseptic
by injections of carbolic acid or other proper agent. Little
need be said as to the purely medicinal treatment. When
there is much fever, aconite, quinine, antipyrine may be given.
In cases occurring in feeble women, especially after childbirth,
alcoholic stimulants may need to be used.
Chronic Metritis.—Fritsch says that “all conditions and
circumstances leading to repeated hyperaemia of the non-
gravid uterus, or preventing the normal puerperal involution
of the organ, are of etiological importance in chronic metritis.”
Goodell says, “ By chronic metritis is meant such tissue
changes in the substance or parenchyma of the womb as are
brought about by a previous inflammation, or from persistent
hyperaemia.” Thomas speaks of the condition as an inflam-
mation. Hart and Barbour state that, “ From a pathological
point of view the term metritis is incorrect, because there
never has been demonstrated a chronic inflammation of the
muscular fiber of the uterus.” Emmet writes that, “ We
look in vain after death for any evidence of metritis or en-
dometritis, or for ulceration of the cervix as it is termed, for
neither of the conditions so-called is inflammatory.” “ When
doctors disagree, who shall decide?” Doctors, evidently;,
but not we.
Included in the term chronic metritis are numerous patho-
logical conditions. If we could approach the subject de novo,
and have the pathological anatomy demonstrated, we could
soon agree upon a name expressive of its pathology, and
upon the proper treatment of the disease. But we do not
know its pathology, absolutely. Nor have we a single disease,
or a single organ, involved. For while in that condition
which we call metritis the parenchyma is most markedly at
fault, we always have pathological states in contiguous tissues
and organs. Fritsch says, “The only facts positively demon-
strated are the enlargement of the uterus, the dilatation of
the vessels, particularly the larger, and the frequently com-
plicating peri, and endo-metritis.”
If we examine a case in its early stage we find the uterus
reddened, soft, sensitive and infiltrated with blood, serum
and fibrin, and consequently enlarged ; and at times altered
in shape. Later on the organ increases still further in size
from a proliferation of connective tissue. As the growth
continues the blood vessels are obliterated or lessened in
size, by the pressure, and the blood supply is diminished.
The organ then becomes anaemic, indurated, and dimin-
ishes in size ; but rarely if ever reaches normal dimen-
sions.
The most common cause of chronic metritis is delayed
involution of the puerperal uterus. The change of muscu-
lar tissue into adipose, and the absorption of the latter, does
not proceed as rapidly as it should.
The organ is hyperaemic, leading to connective-tissue
growth, which remains; while the muscular tissue but slowly
reaches its normal amount. The circulation being inter-
fered with, the organ, from having been too well nourished,
becomes starved. Abortions are oftener followed by metritis
than are labours at term, for now less prudence is observed,
and the uterus is not stimulated to contraction by the nurs-
ing of the child. A piece of retained placenta, a blood-clot'-*
a peri-uterine inflammation maybe an irritation which causes
an hyperasmia and a following metritis.
In the present condition of the uterus pregnancy may
readily take place, to be again followed by abortion or mis-
carriage, and their attendant evils. A lacerated cervix is a
frequent source of metritis. Here we cannot resist using
Emmet’s language: “Laceration of the cervix becomes a
most frequent cause of uterine disease, arresting involution
through the irritation established by the separation of the
flaps. Hypertrophy of the uterus is thus kept up by this
source of irritation, and an intractable form of erosion ap-
pears, with an increased menstrual flow, and an exhausting
leucorrhoea in the intervals. We find prolapse of the
vaginal wall taking place when the proper support is want-
ing at the outlet ; the uterus then becomes retroverted, and,
in consequence of the obstruction to the circulation from its
mal-positicen, it increases still more in size.” Again he says,
“ I would state that, for many years past, I have met with
few or no cases of sub-involution which were not due to
laceration of the cervix.”
If the patient get up too soon after confinement, and what
is “ too soon ” for one may not be for another, the yet large
uterus assumes a malposition, interferes with the circulation,
and a formative congestion results. Imprudences at the
menstrual period, which increase or prolong the normal
congestion, displacements, tumors, excessive sexual excite-
ment, diseases of the liver or heart, or anything which inter-
feres with the pelvic circulation, or determines too great a
uterine blood-supply, may be causes of metritis.
The disease is usually insidious in its development, and the
patient can hardly tell when she first considered herself sick.
She has, perhaps, never felt well since some confinement. Het-
back is weak or aching. Pain extends down the lower limbs,
and they seem to have lost much of their strength. There
is a sense of pressure and weight in the pelvis, and she thinks
she has “falling of the womb.” And she is right. Her general
health fails, a uterine headache distresses her, her appetite is
variable, her bowels usually constipated, her circulation bad.
At first she may repeatedly become pregnant, and abort, and
later become sterile. An endometritis gives her a leucorrhoea,
the character of which varies with the severity of the disease,
and whether the neck or body is chiefly involved.
Physical examination of a patient with metritis involving
the whole organ, which is unchanged by flexions or misplace-
ments of any kind, shows the uterus uniformly enlarged, but
of quite normal shape, but differing from an enlargement due
to pregnancy, inasmuch as the latter has an antero-posterior
thickening, which one, with an uneducated finger, or, on care-
less examination, is apt to diagnose anteflexion. As we are
apt to have flexion, version and prolapse in varying degrees,
neighboring inflammatory deposits and tumors, the uterus
will be found in all shapes, and an accurate diagnosis is often
made with difficulty. The differential diagnosis we pass, hav-
ing incidentally referred to the conditions with which metritis
is most likely to be confounded.
The prognosis is unfavorable, that is, we are rarely able to
restore the organ to its normal condition. If the case is seen
early, if there are no serious complications, and if the patient
be an obedient one, the result of treatment may be so favorable
as to be considered a cure. But usually, when the patient
comes to us, the concurrent troubles are so serious, the struct-
ural alterations so great, that we can only hope to relieve.
Yet, even then, it is in many cases possible to so relieve that
the patient considers herself well. Many cases resist all
treatment. Again the patient disobeys the most judicious
advice, and relapses and exacerbations occur, which are dis-
couraging to all. The great length of time required to effect
a cure, or even an amelioration, taxes the patient’s confidence
as to the final outcome to the utmost, and very often she
drops out of sight, to live on a nervous, irritable invalid? shat-
tered in body and possibly in mind, whose only hope is to be
relieved by nature, when the menopause is reached, and the
uterus undergoes senile atrophy.
Hart and Barbour, in their “ Manual of Gynaecology,” say
that “ chronic metritis is the most important ol all the diseases
of women.” Thomas says, in his treatment of inflammation
of the neck of the uterus, that it “may be regarded as the
most important subject in a work on diseases of women.” Of
course, it is not to be inferred- that a metritic womb is more
important or serious than a carcinomatous one, or than an
ovarian tumor, but that the sum total of evil resulting from
the former is greater than from any other one of the diseases
of women. Hence the great importance of the treatment of
these cases. As already seen, they are seldom uncomplicated.
So we have more than one diseased structure to consider
in applying our remedial measures, and the treatment of
each case must vary as its pathology varies. Concerning
the general treatment there is but little difference of*opinion:
regarding the local there is the greatest variance. Some,
notably among them Dr. Emmet, would trust very largely
to general treatment. He would watch the pelvic circula-
tion, correct malpositions of the organ, and look after gen-
eral nutrition; all of which is certainly of the utmost impor-
tance.
Emmet writes: “As long as we continue to treat the
recognized pathological changes in the uterus as the primary
condition, it will seem proper to apply agents directly, or as-
nearly so as possible, to the parts involved. If, however, we
appreciate the fact that some inflammatory change in the
pelvic tissues, outside the uterus, is existing, or has existed,
in almost every instance of so-called uterine disease in the
non-puerperal state, we shall not attempt to treat the effect
by agents introduced into the cavity of the uterus, without
being prepared for recurrent attacks of cellulitis, excited by
our mode of practice, and not due to some accidental cause.”
Again: “Inflammation of the lining membrane of the
uterine canal does exist under exceptional circumstances as
a distinct condition, but it is rare. In practice, it is believed,
we chiefly find the congestion and supersecretion as an
effect, of little consequence in itself until it has been aggra-
vated by misapplied local treatment.” He also says: “When
an erosion has been healed by caustic applications, the health
of the woman improves rapidly, since, for a time, a great
leak has been stopped, by which she was constantly pouring
out her life-blood in the form of leucorrhoeal discharge. But,
as the primary cause is not removed, the erosion must return
again and again, until at length, if the treatment is continued,
every mucous follicle will have been destroyed, and no fur-
ther discharge can take place; but the hypertrophy of the
uterus and the abnormal condition of the pelvic circulation
will remain. If the application is strong enough to produce
a slough, then, of course, the mucous follicles will be de-
stroyed; but even if milder means, as the use of the nitrate
of silver, are persevered in long enough to heal the surface,,
the damage will be quite as great, since the tissues will have
been rendered sufficiently dense to cause atrophy of these
follicles, and, after either mode of treatment, the tissues
become essentially cicatricial in character.” “ Yet conscien-
tious men of our day, after the use of the cautery or caustics,
will leave a surface on the vagina or cervix to heal by gran-
ulation, and will deny that the surface thus formed is cica-
tricial or that it ever contracts; and I have no reason to
doubt that they think so, but I do impugn the accuracy of
their observation, and the wisdom of their measures.”
On the other hand, the great body of gynaecologists believe
in the importance of local treatment, of course, according to
Emmet, because they know no better. Goodell well repre-
sents this class. He writes that “ while constitutional reme-
dies are not neglected, the chief treatment should be a local
one.” In speaking of caustic applications, he says of nitric
acid, “ I am very partial to this escharotic, nor have I found
that its use is followed by symptoms more urgent than those
produced by the milder caustics. It has been urged that nitric
acid will so far modify or injure the endometrium as to prevent
conception. To this I can reply that I have twice by its
use cured sterility, and that I have repeatedly seen pregnancy
occur in women treated with it.” Even if, after the use of so
powerful an agent as nitric acid, or the repeated use of the less
potent silver nitrate, the mucous membrane does become, or
is changed into, cicatricial tissue, if the diseased condition for
which it is used is essentially cured, and the membrane is in
so normal a condition as to be compatible with pregnancy, the
probability is that the profession will continue to follow these
barbaric methods. And even if, instead of sometimes chring
sterility they so affected the parts that they always caused it,
they would continue to be used, if they transformed the patient
from a sufferer and a cripple to a comparatively well woman.
And it is even possible that it would be a better way of causing
sterility than by removing the ovaries, which it has been pro-
posed to do for the disease under consideration.
As to the greater importance of general or local treatment,
no conclusion can be reached. Each is supplemental to the
other. It is evident that the general treatment is more often
neglected. It is also true that in young and unmarried patients
it should be first faithfully tried.
We find we have two classes of patients to deal with ; to one
of which we have to apply the curb, and to the other the spurs.
In one class we find those naturally ambitious and reluctant
to acknowledge they are half as ill as they are; and also those
whose lots have fallen in harder places, and who are compelled
to work, sick or well, as long as they can stand. In the other
class we have those who naturally have less energy, and who
readily yield when the struggle comes. Here we find many
of the wealthy, and those of whom it is often uncharitably
said, “ they love to be sick.” The advice, and much of the
general treatment, which would be proper for one class would
be ruinous to the other.
All measures calculated to improve the whole system should
be adopted, as tending to improve the uterus as a part of the
whole. A change of residence for a time, giving a change of
scene and air, and a rest to mind and body, is of the greatest
value. The diet, bathing and clothing must receive our atten-
tion, and the intestines be thoroughly unloaded and kept in a
soluble condition. Exercise and rest must be judiciously com-
bined. Suitable tonics, foremost among them iron, will be
needed, and ergot will often help us wonderfully. Alterative
medicines sometimes seem of value. All measures and medi-
cines tending to regulate the pelvic and general circulation
must be carefully attended to.
In our local treatment we must constantly keep in mind the
various and varying conditions our patients present, and as
one or the other predominates, and we change our treatment
accordingly, so will our success be.
We suppose we have a patient with a chronic metritis in-
volving the whole organ, and accompanied as usual by an
endometritis.
We shall probably begin our local treatment by ordering
a hot water vaginal douch, taken once or twice a day. One
or two gallons of water to be taken from a fountain syringe,
and from twenty to thirty minutes to be consumed in^taking
it. In ordering these injections it must not be assumed that
the patient knows anything about them, but minute directions
for their use must be given. About once in three days, we
shall make an application to the entire uterine cavity, of
iodized chloral-phenol, or other mild caustic or astringent.
If the uterine canal is not sufficiently open to permit us to
readily make the application, it must be dilated, preferably
with a uterine dilator. A glycerine tampon' may now be
introduced into the vagina and allowed to remain from
twelve to twenty-four hours. If thought best it may be
medicated, but its chief use is of course to deplete. It may
be made of absorbent cotton, oakum or antiseptic wool. If
there is a misplacement of the uterus an attempt may be
made to use the tampon for a pessary. If this cannot be
done the uterus must be kept in place by a proper pes-
sary, if such can be tolerated. If there are peri-uterine
inflammations, thickenings or adhesions, the vagina and
abdomen may be painted with iodine.
Although pregnancy so frequently results in abortion,
sometimes it is a means of an essential cure. If the patient,
Aneurism, Compiled for the Rock River
ALL DOUBTFUL
So far as possible each Reporter is credited with his own series.
■ L H J O1	< lUTT i TH
o w	-S .a .S -5 s S t ’-5 .2-S o O ° o	>> t: •	-
d q	c x
r*	♦-’Jl	bi	■*-’	do	C	C o	u	*-•	m	r* O	X	o	—<	Q	o	b	i
2	£	oo	rf	£	o	£	5 73	-u	ti	T	£	o	i
O X	X	O	c/5	c/)	2	£ D	D	X	O	O	o “	C	X	2	-*	Z
________________y <1	<1 H •<	<! < H BQ Q B B B B ■<	«$ M o £ |C
REPORTER
Barwell....................................................... I.......... ,...	n.............I
Ciniselli.......... 37........................................ 37........ .........................
■
Crisp....................  59	8--- 98----- 48-------- 21 ....	59 175 --- 18	1	25----
Ehrmann ...........................................         r	.........- ............ 213_____...
Fischer...............................................................     ....	..................
Goodhart...........................................................................           ...
Hayden............... 92..........    _.......1............... 92.........................    ...
Hodgson...............,__	8	21................................. 8	21	...,____ 2_______...
Holmes..................... 2...................................... 2----- ---- 5..........   ...
Kinlock.................................................................. ......................
Koch........................................................................... 79 ...
Lebert...............I__6i03 ................................. I___ 103.........................
I.e Fort............................................................................... 241________
Eisfranc____________________________- ..................   ..	......’. ........     -	...... ..
Meyers...................... 34.......... 75........................... 109	__.... ...	-- --------
Norris.........................................-........................  |____ 60 ....	138}-----
Pilz........................-.......................................................... 4S2 .......
Poore....................... 8........................................... S	______________________
Roux..................................................................... :..._____.... ... |___
Poland...................................................................'......................
Sibson.................. 6i77____ 87 193 140 120 2c 72	71 .... 177 703 _.... .........  ..
Syme........................-........-........................ ........................ ........
Wyeth...........................•............................. ...-I............... ... 789 .... |.
Wenzel 7 .._____________ 7	4---- 2	1	1-------—-----1---- 7	8| ;	1,	11 — ---- 2 --
1 I	I | I [1	1 I	11
1.	Including Wounds.
2.	Femoral, Popliteal and Brachial; group could not be satisfactorily separated.
3.	Also, involving Carotid and Subclavian Artery.
4.	Also, involving Axillary Artery.
5.	Three, near Bifurcation of Arota.
6.	131, near Caeliac Axis.
M edical Society from Various Sources.
ASES OMITTED.
The cases added by myself have never before been tabulated.
2 Jo	'5	.	d 73
r? U	Soo,	2 'S
J-	L»	Ci rt	.	. n
jo	«73	2 2 .13 i • £	§ I p t - 1
I	5	I	I	I I	1	|	I	I	f	f	= |	|	g £	§	I	S	$	*
H	o	2	5	J2 J2	<u	5	•*	£	cl	os	"S.’S	■§	c	o	JS	«5	o	j
g	0.	U	00	K	O	| W	5	|	«	|	O	cl ct	Bi	|	cn cn	m	I	<	cl	0	P>	Z
--------------------------. ______.__________.
. 141 ..............7. 43----.........25--.- 148....... 9...._______i_....1 I22.... 40S
...................................................................................... _...............................................................................2 26 ..	 2	__ 813.     8	86
	 66	1	2	 2.9_ 20	1 137....2. 23.1	 2	- .... 551
......................................................................................-.....................................................................................-....................................................................................-	 213
......................................................................................-   33-.	 S9	    122
3	1......!..............-.........................................-............... 4
.........:.........	 ........... 92
I..................................-	'5.-. 12.......-..... 45...............-..... 63
7
....	.............................................................. 22..........i	22
1
•.......-.................;............I	79
...|.......................................................................        103
........................-
25	........................ 59..................-•...............   95	179
■..................-.................. -....-.................-......... -1.......-	109
--- --.....—-.I....1.................  I......................................     198
■■■........ ■ *
____	... __ I.....................     I	. ... -........... I........... I........-	12
....	............. ..... 27..................................     5	32
•i.............................................. 22..............................   22
. ...............................................................880
i.l............!..................  ........	18...............................      18
KJ.........:........L--..........-------	I—. ----------.......................... 789
1	................. Ill__	1.......... 82______' I 1	11................... 37
I	I	_ J _______I I I_____________I I I "I_______________________
7.	These cases, including my own of dissecting aneurism, have been reported in the Medical
Literature from January to October 14, 1886 ; they are not tabulated elsewhere ; reported by Caton, Hall,
Morris, Styan, Shattuck, A. A. Smith, Berenyi, Black, Sennand, Benham, J. A. Kelly, Prewitt, Cayley,
Barwell, Hobson, Walker and others.
8.	One of these two cases, a man sixty-three years old, a hard drinker, was to he operated on (1S74),
but he tried to get out of his bed contrary to orders, the aneurism burst, gangrene rapidly set in and the
leg was amputated in middle of thigh, with fatal hemorrhage two days later. The case was never reported.
H. P. WENZEL. M.D.
by the greatest care and prudence, escapes the abortion, and
after confinement strictly obeys her physician, she may find
herself with a degree of health unknown to her for a long
time.
3225 Vernon avenue.
				

